# Supplementary material for: Identification of Freezing-Responsive microRNAs and Their Targets in Chinese Jujube by Small RNA and Degradome Sequencing
Source: Int J Mol Sci. 2026 Sep 4;27(17):7912. doi: 10.3390/ijms27177912 (PMC13566890; doi:10.3390/ijms27177912)
Supplement: Supplementary file 1 [file ijms-27-07912-s001.zip › Supplementary Material S2/novel_miR_160.pdf]

Provisional ID	:	novel_miR_160
Score total	:	0.6
Score for star read(s)	:	-1.3
Score for read counts	:	0
Score for mfe	:	0.3
Score for randfold	:	1.6
Score for cons. seed	:	
Total read count	:	3992
Mature read count	:	3972
Loop read count	:	0
Star read count	:	20

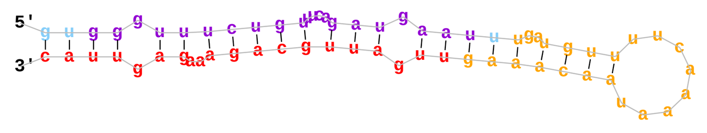

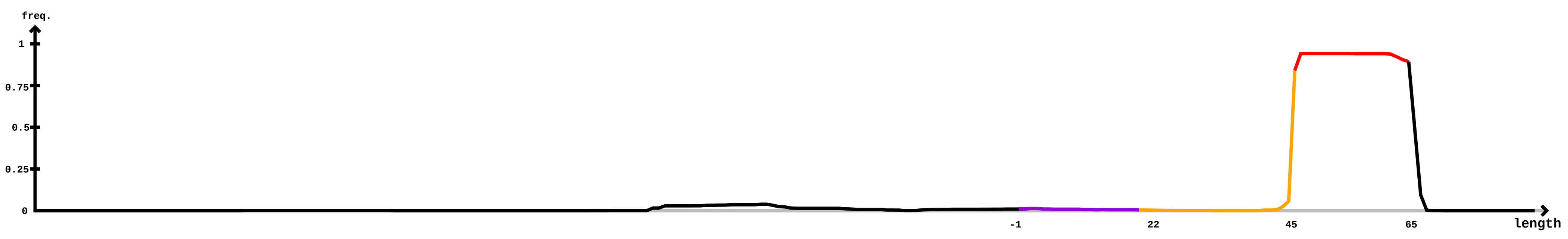

Star Mature

```
5'- ggaagccauuaucaaguuguuauuaucggaugaaauuaguggcaacaagauaucuguaauuccaauaguugggaugggguggaguugggaaaacuacccuugcucaacucguuuauaaggauaucgaugauagggucaugaauaaaccuuuugauuucaaaacguggguuucuguuucagaugaauuugauguuuucaaaauaacaaaguugauugcagaaagaguuacaucccaaauaauugaggucaaa -3'  obs
    ggaagccauuaucaaguuguuauuaucggaugaaauuaguggcaacaagauaucuguaauuccaauaguugggaugggguggaguugggaaaacuacccuugcucaacucguuuauaaggauaucgaugauagggucaugaauaaaccuuuugauuucaaaacguggguuucuguuucagaugaauuugauguuuucaaaauaacaaaguugauugcagaaagaguuacaucccaaauaauugaggucaaa       exp
    ((((..((.((((..((((((((((..........))))))))))..)))).))...))))........((((((((.((((.(((........))).)))).))))))))..((((.(((..(((((...)))))..))).)))))))((((((((((....(((((((((((....(((.(((((..((((........))))))))).))))))))))).........))))....)))))))))).      reads  mm  sample
    .................................aauuaguggcaacaagauaucugua................................................................................................................................................................................................  2  0  JT-2
    .................................aauuaguggcaacaagauaucugua................................................................................................................................................................................................  2  0  JT-3
    ...........................................................................................acuacccuugcucaacucgu...........................................................................................................................................  1  0  LT-3
    ..............................................................................................acccuugcucaacuUguuu.........................................................................................................................................  1  1  LT-1
    .....................................................................................................cucaacucguuuauaaggau.................................................................................................................................  7  0  LT-3
    .....................................................................................................cucCacucguuuauaaggau.................................................................................................................................  2  1  LT-2
    .....................................................................................................cucaacucguuuauaaggau.................................................................................................................................  1  0  JCK-2
    .....................................................................................................cucaacucguuuauaaggau.................................................................................................................................  5  0  LCK-3
    .....................................................................................................cucaacucguuuauaCggau.................................................................................................................................  1  1  LT-2
    .....................................................................................................cucaacucguuuauaaggau.................................................................................................................................  1  0  JCK-1
    .....................................................................................................cucaacucguuuauaaggau.................................................................................................................................  2  0  JCK-3
    .....................................................................................................cucaacucguuuauaaggau.................................................................................................................................  7  0  LT-2
    .....................................................................................................cucaacucguuuauaaggau.................................................................................................................................  1  0  LT-1
    .....................................................................................................cucaacucguuuauaaggauU................................................................................................................................  1  1  LT-1
    .....................................................................................................cucaacucguuuauaaggaua................................................................................................................................  4  0  LT-1
    .....................................................................................................cucaacucguuuauaaggaua................................................................................................................................  8  0  JCK-3
    .....................................................................................................cucaacucguuuauaaggauU................................................................................................................................  2  1  LT-2
    .....................................................................................................cucaacucguuuauaaggaua................................................................................................................................  2  0  LT-3
    .....................................................................................................cucaacucguuuauaaggaua................................................................................................................................  7  0  JCK-1
    .....................................................................................................cucaacucguuuauaaggaua................................................................................................................................  4  0  JCK-2
    .....................................................................................................cucaacucguuuauaaggauU................................................................................................................................  2  1  LCK-3
    .....................................................................................................cucaacucguuuauaaggauUu...............................................................................................................................  3  1  LCK-3
    .....................................................................................................cucaacucguuuauaaggauUu...............................................................................................................................  1  1  LT-3
    .....................................................................................................cucaacucguuuauaaggauauc..............................................................................................................................  1  0  JCK-1
    .....................................................................................................cucaacucguuuauaaggauauc..............................................................................................................................  3  0  JCK-3
    .......................................................................................................caacucguuuauaaggauU................................................................................................................................  4  1  LCK-3
    .......................................................................................................caacucguuuauaaggaua................................................................................................................................  4  0  LT-2
    .......................................................................................................caacucguuuauaaggaua................................................................................................................................  3  0  LT-3
    .......................................................................................................caacucguuuauaaggauUu...............................................................................................................................  1  1  LT-1
    .......................................................................................................caacucguuuauaaggauUu...............................................................................................................................  2  1  LT-2
    .......................................................................................................caacucguuuauaaggauUu...............................................................................................................................  1  1  JCK-1
    .......................................................................................................caacucguuuauaaggauauU..............................................................................................................................  1  1  LCK-3
    .......................................................................................................caacucguuuauaaggauauc..............................................................................................................................  1  0  JCK-3
```

Star Mature

```
ggaagccauuaucaaguuguuauuaucggaugaaauuaguggcaacaagauaucuguaauuccaauaguugggauggguggaguugggaaaacuacccuugcucaacucguuuauaaggauaucgaugauagggucaugaauaaaccuuuugauuucaaaacguggguuucuguuucagaugaauuugauguuuucaaaauaacaaaguugauugcagaaagaguuacaucccaaauaauugaggucaaa
.......................................................................................................caacucguuuauaaggauauU..............................................................................................................................   2   1   LT-3
.......................................................................................................caacucguuuauaaggauauU..............................................................................................................................   7   1   JT-1
.......................................................................................................caacucguuuauaaggauauc..............................................................................................................................   11  0   LCK-3
.......................................................................................................caacucguuuauaaggauauc..............................................................................................................................   3   0   LT-1
.......................................................................................................caacucguuuauaaggauauc..............................................................................................................................   2   0   LT-2
.......................................................................................................caacucguuuauaaggauauU..............................................................................................................................   1   1   LT-1
.......................................................................................................caacucguuuauaaggauauc..............................................................................................................................   5   0   LT-3
.......................................................................................................caacucguuuauaaggauauc..............................................................................................................................   1   0   JCK-1
.......................................................................................................caacucguuuauaaggauaucg.............................................................................................................................   3   0   JCK-2
.......................................................................................................caacucguuuauaaggauaucg.............................................................................................................................   1   0   LT-1
.......................................................................................................caacucguuuauaaggauaucg.............................................................................................................................   1   0   LT-3
.......................................................................................................caacucguuuauaaggauaucgaugauagg.....................................................................................................................   1   0   LT-2
........................................................................................................aacucguuuauaaggauauc..............................................................................................................................   1   0   JCK-2
............................................................................................................cguuuauaaggauaucgaugauagg.....................................................................................................................   1   0   LT-1
.............................................................................................................guuuauaaggauaucgaugauagg.....................................................................................................................   1   0   LT-1
..............................................................................................................uuuauaaggauaucgaugauagg.....................................................................................................................   7   0   LCK-3
..............................................................................................................uuuauaaggauaucgaugauaggU....................................................................................................................   3   1   JCK-2
..............................................................................................................uuuauaaggauaucgaugauaggU....................................................................................................................   2   1   JCK-3
..............................................................................................................uuuauaaggauaucgaugauaggU....................................................................................................................   1   1   JCK-1
...............................................................................................................uuauaaggauaucgaugauagggu...................................................................................................................   1   0   LT-2
................................................................................................................uauaaggauaucgaugauagg.....................................................................................................................   2   0   JCK-3
................................................................................................................uauaaggauaucgaugauagg.....................................................................................................................   1   0   JCK-2
................................................................................................................uauaaggauaucgaugauaggguc..................................................................................................................   1   0   LT-3
.................................................................................................................auaaggauaucgaugauagggu...................................................................................................................   1   0   JCK-2
.................................................................................................................auaaggauaucgaugauagggu...................................................................................................................   1   0   JCK-3
..................................................................................................................uaaggauaucgaugauagggu...................................................................................................................   2   0   JCK-1
..................................................................................................................uaaggauaucgaugauagggu...................................................................................................................   2   0   LCK-3
..................................................................................................................uaaggauaucgaugauagggu...................................................................................................................   1   0   LT-3
..................................................................................................................uaaggauaucgaugauagggu...................................................................................................................   1   0   JCK-2
..................................................................................................................uaaggauaucgaugauagggu...................................................................................................................   1   0   JCK-3
...................................................................................................................aaggauaucgaugauagggu...................................................................................................................   1   0   LT-3
.......................................................................................................................auaucgaugauagggucauga..............................................................................................................   1   0   JCK-3
.......................................................................................................................auaucgaugauagggucauga..............................................................................................................   3   0   LT-3
.......................................................................................................................auaucgaugauagggucauga..............................................................................................................   2   0   LT-1
.......................................................................................................................auaucgaugauagggucauga..............................................................................................................   7   0   LT-2
..........................................................................................................................ucgaugauagggucaugaa.............................................................................................................   1   0   JCK-3
..........................................................................................................................ucgaugauagggucaugaau............................................................................................................   1   0   LT-1
..........................................................................................................................ucgaugauagggucaugaauU...........................................................................................................   1   1   LT-3
..........................................................................................................................ucgaugauagggucaugaaua...........................................................................................................   1   0   LT-1
..........................................................................................................................ucgaugauagggucaugaaua...........................................................................................................   3   0   LT-2
...........................................................................................................................cgaugauagggucaugaau............................................................................................................   1   0   LT-3
............................................................................................................................gaugauagggucaugaaua...........................................................................................................   1   0   LT-3
............................................................................................................................gaugauagggucaugaaua...........................................................................................................   4   0   LT-2
............................................................................................................................gCugauagggucaugaaua...........................................................................................................   1   1   LT-2
............................................................................................................................gaugauagggucaugaaua...........................................................................................................   1   0   LT-1
............................................................................................................................gaugauagggucaugaauaaa.........................................................................................................   1   0   JCK-2
............................................................................................................................gaugauagggucaugaauaaa.........................................................................................................   1   0   JCK-1
...............................................................................................................................................aaccuuuugauuucaaaacgu......................................................................................   1   0   LT-1
.................................................................................................................................................ccuuuugauuucaaaacguggguuucug.............................................................................   4   0   JCK-2
.................................................................................................................................................ccuuuugauuucaaaacguggguuucug.............................................................................   1   0   LT-3
.................................................................................................................................................ccuuuugauuucaaaacguggguuucuguu...........................................................................   1   0   LT-2
..................................................................................................................................................cuuuugauuucaaaacgugU.....................................................................................   2   1   LT-3
..................................................................................................................................................cuuuugauuucaaaacgugU.....................................................................................   12  1   JT-1
..................................................................................................................................................cuuuugauuucaaaacguggguuucuguu...........................................................................   1   0   JCK-2
...................................................................................................................................................uuuugauuucaaaacgugggu..................................................................................   2   0   JCK-2
...................................................................................................................................................uuuugauuucaaaacgugggu..................................................................................   1   0   JCK-1
...................................................................................................................................................uuuugauuucaaaacgugggu..................................................................................   2   0   JCK-3
....................................................................................................................................................uuugauuucaaaacguggguuucug.............................................................................   3   0   LT-2
.....................................................................................................................................................uugauuucaaaacguggguuucug.............................................................................   1   0   JCK-3
.....................................................................................................................................................uugauuucaaaacguggguuucug.............................................................................   1   0   JCK-2
.......................................................................................................................................................gauuucaaaacguggguuucuguu...........................................................................   2   0   LT-3
.......................................................................................................................................................gauuucaaaacguggguuucuguu...........................................................................   1   0   LT-1
............................................................................................................................................................caaaacguggguuucuguuuc.........................................................................   1   0   JCK-2
..............................................................................................................................................................aaacguggguuucuguuuc.........................................................................   2   0   LT-3
................................................................................................................................................................acguggguuucuguuucagaug....................................................................   1   0   JCK-1
................................................................................................................................................................acguggguuucuguuucagaug....................................................................   2   0   JCK-2
...................................................................................................................................................................uggguuucuguuucagauga...................................................................   1   0   LT-3
....................................................................................................................................................................ggguuucuguuucagaugaau.................................................................   1   0   JCK-3
....................................................................................................................................................................ggguuucuguuucagaugaau.................................................................   2   0   JCK-2
....................................................................................................................................................................ggguuucuguuucagaugaau.................................................................   1   0   LT-3
```

Star | Mature

ggaagccauuaucaaguuguuauuaucggaugaaauuaguggcaacaagauaucuguaauuccaauaguugggaugggugggaguugggaaaacuacccuugcucaacucguuuauaaggauaucgaugauagggucaugaauaaaccuuuugauuucaaaacguggguuucuguuucagaugaauuugauguuuucaaaauaacaaaguugauugcagaaagaguuacaucccaaauaauugaggucaaa

ggguuucuguuucagaugaau	3	0	LT-1
ggguuucuguuucagaugaau	4	0	LCK-3
ggguuucuguuucagaugaau	1	0	JCK-1
ggguuucuguuucagaugaauuug	3	0	LT-3
ggguuucuguuucagaugaauuuga	1	0	JCK-2
cagaugaCuuugauguuuu	1	1	LT-1
cagaugaCuuugauguuuu	3	1	LT-2
aaauaacaaaguugauugcag	1	0	LT-1
uaacaaaguugauugcag	1	0	LT-1
caaaguugauugcagaaagag	2	0	JCK-2
caaaguugauugcagaaagag	3	0	JCK-1
caaaguugauugcagaaagag	4	0	JCK-3
caaaguugauugcagaaagag	1	0	LCK-3
caaaguugauugcagaaagagu	1	0	JCK-3
caaaguugauugcagaaagagu	3	0	JCK-2
aaguugauugcagaaagagu	1	0	JCK-2
aaguugauugcagaaagagu	1	0	JCK-3
aaguugauugcagaaagaguu	2	0	LT-3
aaguugauugcagaaagaguu	1	0	LT-1
aaguugauugcagaaagaguu	1	0	JCK-3
aaguugauugcagaaagaguu	3	0	JCK-2
aguugauugcagaaagagu	3	0	LT-3
aguugauugcagaaagagu	4	0	LT-2
aguugauugcagaaagagu	1	0	LT-1
aguugauugcagaaagaguu	2	0	JCK-3
aguugauugcagaaagaguu	3	0	LT-2
aguugauugcagaaagaguu	1	0	JCK-2
aguugauugcagaaagaguu	8	0	LCK-3
aguugauugcagaaagaguu	17	0	JT-1
aguugauugcagaaagaguu	2	0	LT-1
aguugauugcagaaagaguu	6	0	LT-3
aguugauugcagaaagaguua	2	0	JCK-2
aguugauugcagaaagaguua	6	0	JCK-1
aguugauugcagaaagaguua	5	0	JCK-3
aguugauugcagaaagaguua	2	0	LT-3
aguugauugcagaaagaguua	4	0	LT-2
aguugauugcagaaagaguua	10	0	LCK-3
guugauugcagaaagagu	13	0	LCK-2
guugauugcagaaagagu	10	0	LCK-1
guugauugcagaaagagu	8	0	LT-3
guugauugcagaaagagu	3	0	LT-1
guugauugcagaaagagu	1	0	LT-2
guugauugcagaaagagu	16	0	JT-1
guugauugcagaaagagu	2	0	JCK-3
guugauugcagaaagagu	1	0	JCK-1
guugauugcagaaagaguu	1	0	LT-1
guugauugcagaaagaguu	4	0	JCK-1
guugauugcagaaagaguua	4	0	LT-1
guugauugcagaaagaguua	4	0	JCK-2
guugauugcagaaagaguua	1	0	JCK-3
guugauugcagaaagaguuac	7	0	JT-1
guugauugcagaaagaguuac	27	0	JCK-2
guugauugcagaaagaguuac	11	0	JCK-1
guugauugcagaaagaguuac	5	0	LCK-3
guugauugcagaaagaguuac	20	0	JCK-3
Cuugauugcagaaagaguuac	1	1	LT-1
guugauugcagaaagaguuaca	2	0	JCK-3
uugauugcagaaagaguu	1	0	JCK-2
uugauugcagaaagaguu	1	0	JCK-3
uugauugcagaaagaguu	7	0	LT-1
uugauugcagaaagaguu	6	0	LT-3
uugauugcagaaagaguu	6	0	LCK-3
uugauugcagaaagaguu	2	0	JCK-1
uugauugcagaaagaguua	1	0	LCK-2
uugauugcagaaagaguua	2	0	LT-1
uugauugcagaaagaguua	1	0	LCK-3
uugauugcagaaagaguuU	3	1	LT-2
uugauugcagaaagaguuU	1	1	LT-1
uugauugcagaaagaguua	1	0	LCK-1
uugauugcagaaagaguua	2	0	LT-3

Star Mature

ggaagccauuaucaaguuguuauuaucggaugaaauuaguggcaacaagauaucuguaauuccaauaguugggaugggguggaguugggaaaacuacccuugcucaacucguuuauaaggauaucgaugauagggucaugaauaaaccuuuugauuucaaaacguggguuucuguuucagaugaauuugauguuuucaaaauaacaaaguugauugcagaaagaguuacaucccaaauaauugaggucaaa			
uugauugcagaaagNguuac	1	1	LCK-1
uugauugcagaaagCguuac	1	1	LCK-3
uugauugcagaaagaguuac	297	0	LT-2
uugauugcCgaaagaguuac	1	1	LT-2
uugCuugcagaaagaguuac	4	1	LT-2
uugauugcagaaagaguuac	48	0	JCK-2
uugauugcagaaagaguuac	462	0	LCK-3
uugauugcagaaagaguuac	247	0	LT-3
uugauugcagaaagaguuac	178	0	LT-1
uugauugcagGaagaguuac	1	1	LT-2
uugauugcagaaagaguuac	9	0	JT-1
uugauugcagaaagaguuCc	1	1	LT-2
uugauugcagaaagaguuac	9	0	JT-3
uugauugcagaaagaguuac	44	0	JCK-1
uugauugcagaaagaguuac	19	0	JCK-3
uugauugcagaaNgaguuac	1	1	LCK-1
uugauugcagaaagaguuac	6	0	JT-2
uugauugcagaaagGguuac	1	1	LCK-3
uugCuugcagaaagaguuac	2	1	LT-1
uugauugcagCaagaguuac	1	1	LT-3
uugauugcagaCagaguuac	1	1	JT-2
uugauugcagaaagaguuac	212	0	LCK-2
uugauugcagaaagaguuac	216	0	LCK-1
uuUauugcagaaagaguuac	1	1	LCK-2
uuUauugcagaaagaguuac	1	1	LCK-1
uugCuugcagaaagaguuac	1	1	LT-3
uugCuugcagaaagaguuac	2	1	LCK-3
uugauugcagaCagaguuac	1	1	JCK-2
uugauugcagaaagaguuaca	137	0	JCK-2
uugauugcagaaagaguuCca	1	1	LT-2
uugCuugcagaaagaguuaca	2	1	LT-2
uugauugcagaaagaguuaNa	1	1	JCK-1
uugauugcagaaagaguuaca	158	0	LT-2
uugauugcagaaagaguuaca	281	0	LT-1
uNgauugcagaaagaguuaca	1	1	LT-1
uugauugcagaaagaguuaca	103	0	JCK-3
uugauugcagaCagaguuaca	1	1	LCK-2
uugauugcagaaagaguuaca	2	0	JT-3
uugauugcagaaagaguuCca	1	1	JCK-1
uugauugcCgaaagaguuaca	1	1	LT-3
uugauugcagaaagaguGaca	1	1	JCK-1
uugauugcagaaagCguuaca	1	1	LT-1
uugauugcagaaagaguuGca	1	1	LT-3
uugauugcagaaagaguuaca	76	0	LCK-2
uuNauugcagaaagaguuaca	2	1	LT-2
uugauugcagaaCgaguuaca	1	1	LT-3
uugauugcagaaagaguuacU	5	1	JCK-2
uugCuugcagaaagaguuaca	2	1	LCK-3
uugauugcagCaagaguuaca	1	1	LT-3
uugauugcagaaagaguuaca	66	0	LCK-1
uugauugcagaaagaguuacU	1	1	LT-1
uugauugcagaaagaguuaca	97	0	JCK-1
uugauugcagaaagaguuaca	313	0	LCK-3
uugauugcagaaagaguuaca	2	0	JT-2
uugauugcagaaagaguuaca	264	0	LT-3
uugauugcagaaagaguuacau	1	0	LCK-3
uugauugcagaaagaguuacauU	1	1	LT-3
ugauugcagaaagaguuac	4	0	LT-1
ugauugcagaaagaguuac	1	0	JCK-1
ugauugcagaaagaguuac	7	0	LCK-3
ugauugcagaaagaguuac	3	0	LT-2
ugauugcagaaagaguuac	10	0	LT-3
ugauugcagaaagaguuaca	1	0	LT-3
ugauugcagaaagaguuaca	1	0	LT-1
ugauugcagaaagaguuaca	1	0	JCK-2
ugauugcagaaagaguuaca	1	0	JCK-1
ugauugcagaaagaguuaca	2	0	LT-2
ugauugcagaaagaguuacau	23	0	LT-3
ugauugcagaaagaguuacau	36	0	LT-2
ugauugcagaaagaguuacau	4	0	JT-2

```
                                                                                                                                                                                         Star                                           Mature
ggaagccauuaucaaguuguuauuaucggaugaaauuaguggcaacaagauaucuguaauuccaauaguugggauggguggaguugggaaaacuacccuugcucaacucguuuauaaggauaucgaugauagggucaugaauaaaccuuuugauuucaaaacguggguuucuguuucagaugaauuugauguuuucaaaauaacaaaguugauugcagaaagaguuacaucccaaauaauugaggucaaa
.................................................................................................................................................................................................................ugauugcagaaagaguuacau....................	6	0	JT-1
.................................................................................................................................................................................................................ugauugcagaaagaguuacau....................	35	0	LT-1
.................................................................................................................................................................................................................ugauugcagaaagaguuacau....................	7	0	JT-3
.................................................................................................................................................................................................................ugauugcagaCagaguuacau....................	1	1	JCK-2
.................................................................................................................................................................................................................ugauugcagaaagaguuacau....................	4	0	LCK-3
.................................................................................................................................................................................................................ugauugcCgaaagaguuacau....................	1	1	JCK-1
.................................................................................................................................................................................................................ugauugcagaaagaguuacau....................	108	0	JCK-2
.................................................................................................................................................................................................................ugauugcagaaagaguuacau....................	80	0	JCK-1
.................................................................................................................................................................................................................ugauugcagaaagaguuacau....................	83	0	JCK-3
.................................................................................................................................................................................................................ugauugcagaaagaguuacauc...................	2	0	LT-3
.................................................................................................................................................................................................................ugauugcagaaagaguuacauc...................	1	0	LT-2
.................................................................................................................................................................................................................ugauugcagaaagaguuacauG...................	1	1	JCK-3
.................................................................................................................................................................................................................ugauugcagaaagaguuacauc...................	1	0	JCK-3
.................................................................................................................................................................................................................ugauugcagaaagaguuacauc...................	1	0	LT-1
.................................................................................................................................................................................................................ugauugcagaaagaguuacauG...................	1	1	JCK-1
.................................................................................................................................................................................................................ugauugcagaaagaguuacauccc.................	1	0	JCK-1
.................................................................................................................................................................................................................ugauugcagaaagaguuacauccc.................	2	0	JCK-3
```
